# Transporting Blood to Remote Areas: The Impact of Blood Shaker Machine Vibration on the Quality of Erythrocytes, Hemoglobin, and Lactate Dehydrogenase Levels

**DOI:** 10.4314/ejhs.v34i6.10

**Published:** 2024-11

**Authors:** Jeffri Ardiyanto, Jessica Juan Pramudita, M Syamsul Arif Setiyo Negoro, Slamet Wardoyo

**Affiliations:** 1 Health Polytechnic Ministry of Health Semarang, Indonesia; 2 Department of Environmental Health, Poltekkes Kemenkes Surabaya, indonesia

**Keywords:** Blood Quality, Vibration, Blood Shaker Machine, Whole Blood, Blood Transportation

## Abstract

**Background:**

Effective transportation of blood is essential for ensuring accessible and high-quality blood for transfusion. However, vibration can have a negative impact on blood quality, leading to the loss of erythrocytes and hemoglobin. The aim of the study was to analyse the effect of different vibration exposures for 15 minutes on erythrocyte, haemoglobin and lactate dehydrogenase (LDH) levels of blood samples.

**Methods:**

A quasi-experimental study was conducted on blood donors at the Semarang, Central Java, unit vehicle of the Indonesian Red Cross. Blood samples were collected from donors and exposed to different vibration frequencies for 15 minutes. The erythrocyte, hemoglobin, and lactate dehydrogenase (LDH) levels of the blood samples were then measured. Tests were carried out on three treatment groups, namely control, 6 Hz vibration, and 11 Hz with 30 bags of blood tested with each treatment group of 10 samples.

**Results:**

The results showed that vibration had a significant impact on the integrity of erythrocytes and hemoglobin in whole blood The group exposed to 6 Hz vibration had significantly lower erythrocyte levels than the control group and the group exposed to 11 Hz vibration. The hemoglobin levels after treatment also varied significantly between treatment cohorts, with significantly lower hemoglobin levels observed in the 11 Hz vibration group compared to both the control group and the 6 Hz vibration group. In addition, there was a significant difference between the LDH levels of the various groups following treatment.

**Conclusion:**

The findings of this study suggested that vibration can have a negative impact on blood quality, leading to the loss of erythrocytes and hemoglobin. To protect blood product integrity and reduce the risk of transfusion-related losses, it is essential to implement appropriate vibration mitigation strategies during blood product transport.

## Introduction

The archipelagic landscape of Indonesia poses significant logistical hurdles when it comes to the transportation of blood products. Inadequate transportation infrastructure, particularly in remote regions, results in delays and difficulties in delivering blood to hospitals and healthcare facilities. Regrettably, the uneven distribution of blood supply and prevailing shortages across different regions of Indonesia perpetuate limited access to this vital resource, particularly in remote and underserved areas (1). Even under normal circumstances, transportation logistics in navigating remote islands prove arduous (2).

The Indonesian Red Cross (IRC) assumes exclusive control over the blood supply in the country. The IRC employs conventional cooler boxes to regulate blood temperature during transportation (3). Nevertheless, ensuring adequate temperature control, especially in tropical climates, remains a challenge. Current technologies do not provide a foolproof solution for achieving temperature stability or effectively mitigating the impact of vibrations during blood transportation (4). Consequently, concerns regarding the preservation of blood quality during transit continue to persist. Moreover, insufficient storage facilities at blood collection centers, blood banks, and transportation vehicles exacerbate the difficulties in maintaining optimal storage conditions for blood components (5).

High-frequency vibrations and improper temperature settings during shipment can result in detrimental effects on blood cells (6). Notably, alterations in the hue of blood plasma, contingent upon the concentration of hemoglobin (Hb), hold promise as potential biomarkers for tissue injury. Therefore, comprehensive assessment of erythrocyte quantity and quality, hemoglobin levels, and lactate dehydrogenase (LDH) levels assumes paramount importance in ensuring optimal blood quality standards (7). However, the adherence to proper packaging, handling protocols, and comprehensive monitoring and tracking systems during transportation is pivotal to safeguard the safety and security of blood, as inadequacies in these aspects can compromise blood integrity (8).

Previous studies have predominantly focused on the technological aspects of blood transfusions, encompassing donor selection, infectious disease screening, storage procedures, and temperature control (9). Nevertheless, the specific investigation of the influence of vibrations on blood quality during transportation has been limited (10). Studies have demonstrated that blood transported via refrigerated commercial aircraft remains uncompromised, with reduced vibrations compared to land-based transportation modes (11). Furthermore, drones have emerged as a potential transportation method with minimal vibrations, suggesting their feasibility in ensuring blood quality during transit (12).

The necessity for comprehensive research in developing economies, where ground transport constitutes the primary mode of blood delivery (13), underscores the imperative of investigating the impact of vibrations on erythrocyte integrity, hemoglobin levels, and LDH levels in whole blood. Notably, studies have shown that vibrations, particularly those experienced during automobile transportation, can significantly degrade blood quality (14). Vibrations ranging from 6 to 11 hertz are frequently encountered by individuals in their daily routines. It is the frequency range of vibrations produced, for instance, by walking, driving, and operating apparatus. As a result, considerable research has been devoted to examining the impacts of vibration on human health and performance within this particular frequency range. Consequently, a thorough understanding of the multifaceted dynamics involved in blood transportation is indispensable.

## Materials and Methods

A quasi-experimental study was conducted in September–October 2022 at the Semarang Resort Police unit automobile in Central Java to evaluate the effects of vibration on the quality, quantity, shape, hemoglobin, and LDH levels of erythrocytes. Blood samples were collected from donors aged 17–25, weighing at least 45 kg, with a minimum hemoglobin level of 12.5 gr/dl, and having a systolic blood pressure of 110–160 mm Hg and a diastolic blood pressure of 70–90 mmHg. Women who were menstruating, pregnant, or breastfeeding, as well as donors who had taken medication within the past three days, were excluded. A maximum of 30 blood bags were tested. Thirty is a common sample size for quasi-experimental studies. This is because it is large enough to provide statistically significant results, but it is also small enough to be manageable (43). Blood samples were stored for 24 hours at 2-6°C before testing. This storage duration was done to mimic realistic scenarios in the field, where blood needs to be temporarily stored before use or testing. Besides, donating blood can be a time-consuming and physically demanding process. Limiting the number of blood bags collected from each donor helps to minimize the risk of harm to the donor.

We assessed the CBC to evaluate erythrocyte and hemoglobin levels in whole blood. We utilized a Hematology Analyzer, a widely accepted device in clinical settings, for this purpose. The Hematology Analyzer accurately and rapidly measures blood cell counts, including erythrocytes, and determines hemoglobin levels. To assess LDH levels in blood plasma as an indicator of blood quality, we employed a Clinical Chemistry Analyzer. This device is routinely used in clinical laboratories and diagnostic settings. It measures LDH activity in the blood, providing valuable insights into cell damage or hemolysis.

The independent variable was the test tube blood storage combined with vibration. Two different frequencies of vibration were used: 6 Hz and 11 Hz, generated by a Blood Shaker Machine. The treatment groups included Group A (no vibration), Group B (6 Hz vibration for 15 minutes), and Group C (11 Hz vibration for 15 minutes). Each treatment group received blood bag samples for analysis. The effects of vibration on erythrocyte, hemoglobin, and LDH levels in whole blood were assessed using Fisher's exact test for categorical data. Peripheral blood image analysis of erythrocytes before and after treatment was performed to determine the impact of the Blood Shaker Machine on blood quality. The size of erythrocyte cells within typical ranges was examined through peripheral blood analysis. Haematology analyzers were used to measure hemoglobin levels, and clinical chemistry analysis assessed LDH levels in blood plasma as a measure of LDH quality.

The Shapiro-Wilk test was used to assess the normal distribution of erythrocyte, hemoglobin, and LDH values in the control, 6 Hz, and 11 Hz groups. This test helped researchers determine the appropriate statistical procedures to analyze the data accurately. Parametric tests such as t-tests and ANOVA assume normality and can be used to compare means or evaluate group differences if the data follow a normal distribution (p > 0.05). By using the Shapiro-Wilk test, the researchers ensured the selection of suitable statistical methods to derive precise findings regarding the impact of vibration on whole blood variables. The Repeated Measurement ANOVA test (95% CI) was used to identify changes before and after treatment of haemoglobin, LDH and erythrocytes, while differences between treatment groups used One Way ANOVA (95% CI).

## Results

In the study, the Shapiro-Wilk test was used to assess the distribution of post-haemoglobin levels in the 6 Hz vibration group and LDH levels in the 11 Hz vibration group. The p-values for these two groups were less than 0.05, indicating that the data on these variables are not normally distributed. However, the p-values for the erythrocyte, platelet, hemoglobin, and LDH levels in all other treatment groups were greater than 0.05, indicating that the distributions of these variables are normally distributed.

The results of the one-way analysis of variance (ANOVA) test indicated that the pre-treatment erythrocyte levels were homogeneous (p = 0.090). However, there were significant differences in erythrocyte counts across the treatment groups during the post-test phase (p = 0.042). The 6 Hz vibration intervention resulted in a reduction of erythrocyte counts to 4,220,000/mm3, which was the lowest among the control and 11 Hz vibration groups.

A repeated measures ANOVA (RANOVA) was also conducted for each treatment group. The only group that exhibited a significant difference between the pre- and post-tests was the 11 Hz vibration group (p = 0.017). Notably, vibration therapy at 6 Hz led to the greatest decrease in erythrocyte count, reaching 4,220,000/mm3.

The control group had a mean erythrocyte count of 4,880,000/mm3 (range = 3,890,000-6,300,000) and a mean hemoglobin level of 13.96 g/dL (range = 11.50-16.40). The 6 Hz vibration group had a mean erythrocyte count of 4,370,000/mm3 (range = 3,900,000-5,020,000) and a mean hemoglobin level of 13.07 g/dL (range 11.90-15.00). The 11 Hz vibration group had a mean erythrocyte count of 4,205,000/mm3 (range = 3,090,000-5,320,000) and a mean hemoglobin level of 11.52 g/dL (range = 9.50-15.50). Mean erythrocyte count, which is alternatively referred to as red blood cell count (RBC count), falls within the typical range of 4.2 to 5.4 million cells/mm3 for males and 3.9 to 5.6 million cells/mm3 for females.

The homogeneity analysis of the treatment and control groups found that the p-value exceeded 0.05, indicating that the samples were homogeneous.

One-way ANOVA revealed that pre-treatment hemoglobin levels were homogeneous across treatment groups (p = 0.060). However, post-treatment hemoglobin levels varied significantly by cohort (p = 0.015). Specifically, 10 Hz vibration therapy reduced hemoglobin levels to 10.77 g/dL, a significant decrease compared to the control and 6 Hz vibration groups. Repeated measures ANOVA showed that only the 10 Hz vibration treatment group had a significant difference between pre-test and post-test hemoglobin levels (p = 0.010). One-way ANOVA was used to assess pre-treatment LDH levels across treatment groups. The results showed that the pre-treatment LDH levels were homogeneous (p = 0.322). However, post-treatment LDH levels differed significantly by treatment group (p = 0.000). Specifically, the 11 Hz vibration group had the largest increase in LDH levels, reaching 352.53 U/L. This was significantly higher than the levels in the control and 6 Hz vibration groups. A repeated measure ANOVA was used to analyze the pre-test-post-test data for each treatment group. The results showed that only the 11 Hz vibration treatment group had a significant difference between pre-test and post-test LDH levels (p = 0.005) (table 1).

**Table 1:** Baseline and post test statistical analysis change in Hemoglobin, LDH and Erythrocyte

Measurement Time	Control	6 Hz vibration	11 Hz vibration	p*
		Erythrocyte (million/µl)		
Pretest	4.88±0,71	4.37±0.42	3.94±0.71	0.090
Posttest	4.78±3.74	4.22±0.51	3.61±0.74	0.042
p-value**	0.305	0.172	0.017	
		Hemoglobin (million/µl)		
*Pretest*	13.96±1.67	13.07±1.22	11.52±1.76	0.060
*Posttest*	14.05±1.76	13.28±1.44	10.77±1.73	0.015
p-value**	0.279	0.138	0.010	
		LDH (U/l)		
*Pretest*	146.74±25.83	161.53±21.68	150.31±20.32	0.332
*Posttest*	144.29±27.89	164.20±24.83	352.53±142.91	<0.001
p-value**	0.357	0.328	0.005	

Based on research findings, the average erythrocyte count in males ranges from 4.3 to 5.6 million/microliter, while in females, it ranges from 3.9 to 5.1 million/microliter.

**Figure 1:**
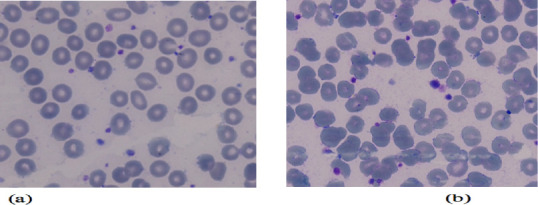
Examination results of the control group for peripheral blood examination (erythrocyte shape)- (a) Pre - Control, (b) Post – Control

**Figure 2:**
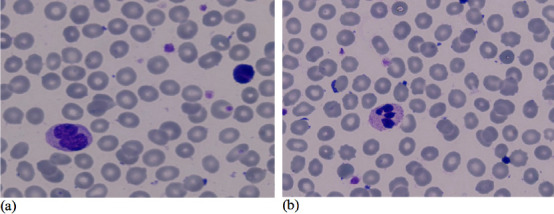
Results of peripheral blood examination (erythrocyte shape) with 6 Hz vibration- (a) 6 Hz vibration - Pre, (b) 6 Hz vibration – Post

**Figure 3:**
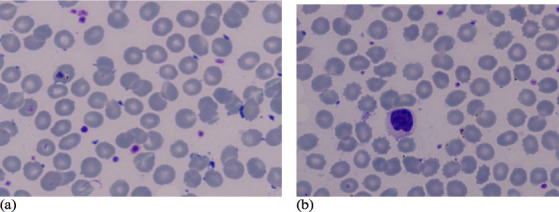
Results of peripheral blood examination (erythrocyte shape) with 11 Hz vibration- (a) 11 Hz vibration, (b) 11 Hz vibration

Giemsa staining as displayed in Figures 1(a), 2(a), 3(a) was used to assess erythrocyte morphology in the control group and the two vibration groups. The results showed that the erythrocytes in the control group were normochromic and normocytic, with a normal range of color and shape. However, some cells with different forms and sizes, such as ovalocytes, stomatocytes, and microcytic cells, were also observed.

Figure 1(b) illustrates that microcytic, normochromic erythrocytes, while Figure 2(b) and Figure 3(b) showed normochromic erythrocytes after the 6 Hz and 11 Hz vibration treatments, respectively. The erythrocytes in the 6 Hz and 11 Hz vibration groups showed some morphological changes, with the most noticeable changes occurring in the 11 Hz vibration group. The erythrocytes in this group were microcytic and normochromic, and some cells were created.

## Discussion

These findings suggest that vibration exposure can lead to changes in erythrocyte formation. The extent of these changes depends on the vibration frequency, intensity, and duration. The results of this study are consistent with previous research that has shown that vibration exposure can damage blood cells and organs. The lifespan of erythrocytes also contributes to the observed changes. Humans typically replace approximately 1% of their erythrocytes daily, and the average count of human erythrocytes ranges from 5,000 to 10,000 x 106/L. This means that the erythrocytes in the vibration groups were likely to have been damaged by the time they were examined.

The findings of this study highlight the importance of considering the vibration threshold when assessing the potential effects of vibration exposure on the body. The results also suggest that the duration and frequency of vibration exposure are important factors to consider. Further research is needed to determine the mechanisms by which vibration exposure causes changes in erythrocyte morphology and to assess the long-term health implications of these changes.

Blood bag haemoglobin changes during transportation and storage in cooler containers may signal quality and integrity issues (15). Erythrocytes carry oxygen via haemoglobin. Hemolysis releases haemoglobin into plasma by rupturing erythrocytes. During shipping and storage, temperature changes, vibration, and rough handling can cause hemolysis (16).

Oxidative stress can also cause haemoglobin changes. Oxidative stress arises when ROS generation outpaces antioxidant defences (17). Reactive oxygen species (ROS) from temperature, storage, or light destroy erythrocytes and release haemoglobin. Oxidative stress and erythrocyte breakdown may cause elevated haemoglobin (18). Haemoglobin degradation or loss may reduce oxygen-carrying capacity, affecting transfusion efficacy and patient outcomes (19). Haemoglobin from damaged erythrocytes increases blood clotting. Red blood cells may rupture more easily during transport and storage if haemoglobin levels are high (20). After long-term vibration at frequencies above 10 Hz, erythrocyte quality deteriorates. Erythrocyte membrane disturbances cause this harm (21).

The lungs function as an organ that absorbs oxygen-carrying hemoglobin from the air and circulates it throughout the body. The average adult blood hemoglobin concentration is about 15.0 g/cm3 and is influenced by age and gender. Mountain dwellers have higher hemoglobin than lowlanders, probably due to their higher altitude homes. This phenomenon is related to the oxygen concentration in the atmosphere, which tends to be lower at higher altitudes (22).

The results of this study suggest that vibration exposure can have a negative impact on LDH levels. The extent of this impact depends on the vibration frequency, intensity, and duration. Erythrocyte viability count and morphology can indicate impairment. LDH is transported by erythrocytes (23). Its catalytic role in the final erythrocyte glycolytic pathway is a key. Erythrocyte hemolysis releases LDH into plasma. Hemolysis immediately increases plasma LDH. LDH may indicate sample preservation hemolysis (24).

LDH levels in blood bags during transport and storage in cooler boxes can reflect blood quality and stability. Hemolysis erythrocyte rupture may cause blood bag LDH levels to rise. Temperature, handling, and vibration can cause hemolysis during transport and storage. Increased LDH levels indicate erythrocyte damage and possible blood quality loss. Oxidative stress can raise blood bag LDH during transport and storage (17).

Red blood cells' ATP levels can alter their activity and physiological activities (25). Erythrocytes run on ATP. ATP depletion disrupts energy metabolism, limiting energy production. This can impair cell activities like membrane integrity and ion and metabolite transport. ATP protects erythrocyte membranes. Membrane fragility and mechanical stress can increase with low ATP levels. ATP deficiency can reduce erythrocyte deformability and tissue oxygen delivery (26). ATP decreases can impair NO control, compromising vascular function and blood flow (27,28).

Poor transportation and storage can decrease the number of erythrocytes, hemoglobin, and LDH in blood, which can impair their function and physiological roles. Therefore, efficient and timely blood transportation requires communication and coordination between blood collection centers, blood banks, hospitals, and transportation providers. Poor communication and coordination can lead to delays, errors, and difficulties in meeting patients' transfusion requirements. To address these issues, it is necessary to improve transit infrastructure, storage facilities, temperature control, coordination, and monitoring and quality assurance programs. To guarantee safe and high-quality blood for transfusion across Indonesia, government authorities, healthcare institutions, and blood transfusion services must collaborate to address these issues.

In conclusion, vibration intensity and erythrocyte damage are positively correlated, meaning that as vibration intensity increases, erythrocyte damage also increases. Vibration also reduces hemoglobin levels in undiluted blood and alters whole blood erythrocyte shape. Higher vibration intensities cause more damage to erythrocytes, and this is reflected in increased whole blood LDH levels.The Blood Shaker Machine is limited to a maximum vibration frequency of 11 Hz, and the test results are only valid for 15 minutes. This means that the machine cannot simulate the full range of vibration frequencies and durations that blood may be exposed to during transportation.

Further research is needed to examine a wider range of vibration frequencies and durations to determine the thresholds for harmful effects and the optimal vibration settings for blood movement. Scientists and healthcare practitioners can improve blood transfusion safety and efficacy by studying the impact of vibration on blood quality and blood transportation practices.

Particular limitations apply to the conclusions drawn in this investigation. Due to its reliance on a 15-minute testing duration and a maximal vibration frequency of 11 Hz, the study's applicability to the entire spectrum of real-world vibration conditions may be limited. It is advisable to exercise caution when generalizing the observed correlation between erythrocyte damage and vibration intensity to different contexts or prolonged exposure periods. In addition, critical hemolysis-related parameters such as plasma-free haemoglobin, haptoglobin, and bilirubin concentrations were not assessed in the study. Such data would have contributed to a more comprehensive comprehension of the effect on blood quality.

This study has several limitations that need to be considered in the interpretation of the results. One major limitation is the use of blood from different bags for various tests, which may affect the variation in results between tests. Although each blood bag comes from a donor who meets the standard eligibility criteria, differences in individual donor characteristics, such as baseline haemoglobin levels, erythrocyte size and LDH levels, may affect the final results. The use of blood from different sources also allows for different responses to the vibrations induced by the *blood shaker* machine, which can be a variable that is difficult to control perfectly. Natural biological variations between donors may have led to more heterogeneous results, thus limiting our ability to conclude definitively on the effect of vibration on blood quality. In addition, the duration of blood storage prior to testing (24 hours) could also have affected the stability of certain blood components, even though storage was performed according to standardised guidelines. This limitation needs to be considered in the context of applying the study results in the field, especially when considering blood transport to remote areas that may take longer. We suggest that future studies consider the use of uniform blood samples or additional tests to address these variations, so that more consistent results can be obtained.
